# Left Distal Radial Artery Access-Site Pseudoaneurysm Treated With Open Surgical Repair

**DOI:** 10.1016/j.jscai.2023.101047

**Published:** 2023-06-10

**Authors:** Ethan C. Korngold, Daniel E. Westerdahl, Brant W. Ullery

**Affiliations:** Providence St. Vincent Medical Center, Providence Heart and Vascular Institute, Portland, Oregon

**Keywords:** access-site complication, distal radial access, pseudoaneurysm, vascular complication

## Case report

A 45-year-old man with a history of hypertension presented with chest pain for 3 hours and electrocardiogram demonstrating ST-elevation myocardial infarction. He was treated with aspirin, heparin, and ticagrelor and underwent primary percutaneous coronary intervention (PCI) of the left anterior descending and diagonal arteries using a 6F catheter and left distal radial artery access. Distal left radial arterial access was obtained using ultrasound guidance per standard protocol. After the procedure, hemostasis was achieved with a PreludeSYNC Distal Radial hemostasis band (Merit Medical) that was applied for 2 hours per hospital protocol. Subsequently, an echocardiogram demonstrated an ejection fraction of 30%, with apical hypokinesis and a 1.9 × 1.5-cm^2^ apical thrombus. The patient was treated with unfractionated heparin and goal-directed medical therapy for heart failure. Then, he underwent a staged PCI of the circumflex artery through right radial artery access; this access site was chosen so as to not reaccess the left radial site. He was discharged on a low-molecular-weight heparin bridge to warfarin for left ventricular thrombus and clopidogrel as a sole antiplatelet agent. His bilateral radial artery access sites revealed no hematomas or masses, and the patient reported no discomfort at the time of discharge, 5 days after his initial presentation and primary PCI.

**Figure 1:**
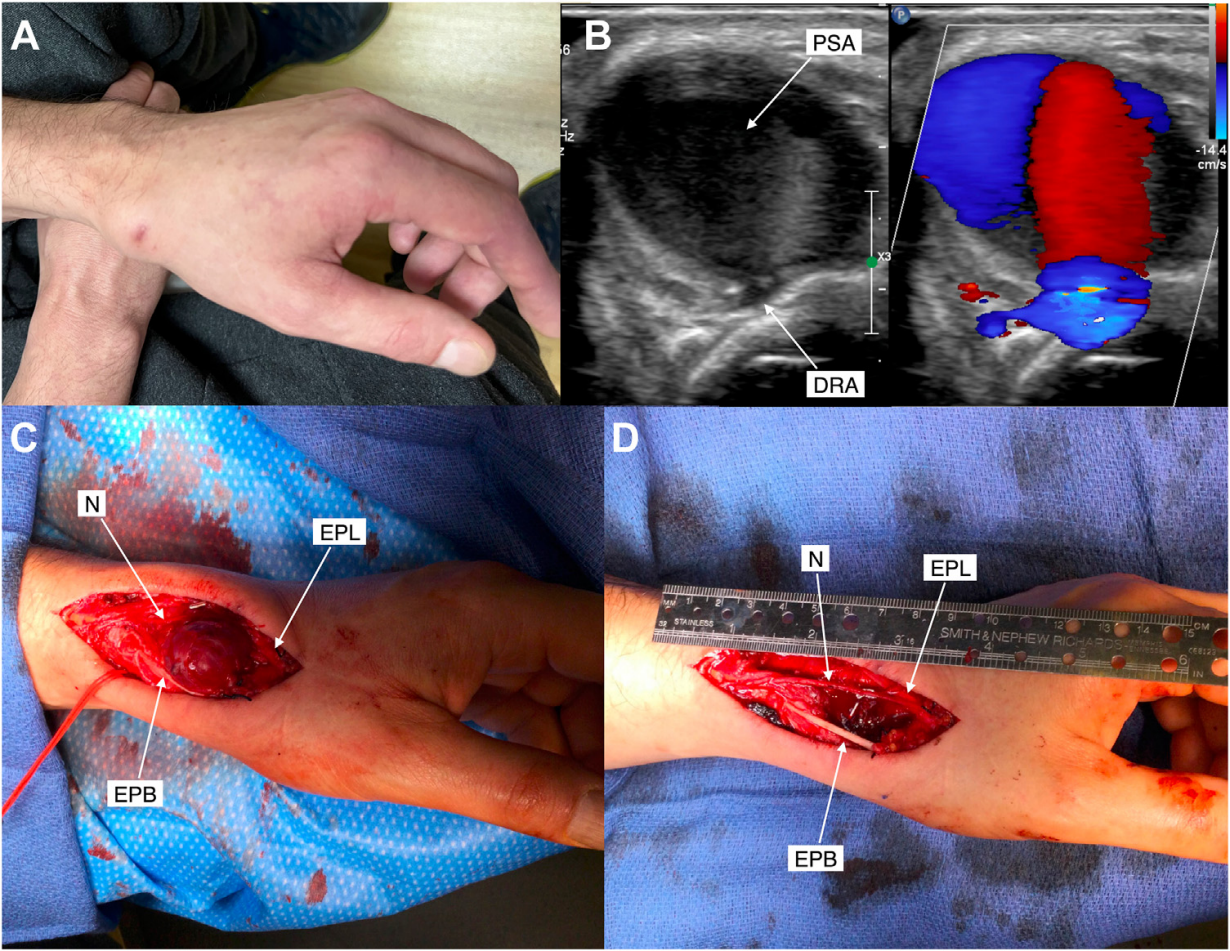
**Distal radial artery pseudoaneurysm.** (**A**) Clinical appearance of the pseudoaneurysm at the left distal radial access site 4 weeks after percutaneous coronary intervention. (**B**) Duplex and Doppler color flow ultrasound of the pseudoaneurysm and distal radial artery. Operative appearance of the pseudoaneurysm (**C**) on exposure and (**D**) after resection, showing a dorsal digital branch of the radial nerve, tendons of the extensor pollicis longus, and the extensor pollicis brevis. DRA, distal radial artery; EPB, extensor pollicis brevis; EPL, extensor pollicis longus; N, nerve; PSA, pseudoaneurysm.

Three days after discharge, the patient resumed a strenuous weightlifting program and noted progressive pain and swelling at the left distal radial access site. On a follow-up examination at 4 weeks, this was observed to be a tender, firm, well-circumscribed pulsatile mass at the left hand anatomic snuffbox (Figure 1A). The perfusion of the left hand was normal on examination, and the patient had no signs or symptoms of local or systemic infection. He did complain of mild numbness in the left palm in the distribution of the superficial branch of the radial nerve. Immediate vascular surgical consultation was obtained. Duplex ultrasound imaging demonstrated a pseudoaneurysm measuring 1.9 × 1.8 cm^2^ with active flow arising from the left distal radial artery (Figure 1B). The patient underwent open surgical repair without interruption of anticoagulation. On surgical exposure, the pseudoaneurysm was apparent within the anatomic snuffbox with surrounding inflammation and tension on the adjacent sensory nerves (Figure 1C). The pseudoaneurysm was resected given its large size, and the distal radial artery was ligated (Figure 1D). Ulnar and palmar arch Doppler signals remained triphasic. The patient recovered well with intact hand function.

## Discussion

Distal radial artery access for percutaneous coronary angiography and intervention is an area of great interest as a refinement of conventional radial artery access with a goal of improved patient comfort, operator ergonomics, and minimizing radial artery complications such as radial artery occlusion. A recent meta-analysis demonstrated that compared with conventional radial artery access, distal radial artery access was associated with lower risks of radial artery occlusion and hematoma.^1^ Pseudoaneurysm is a rare vascular complication of conventional radial artery access, occurring in 0.03% to 0.09% of cases.^2^ The risk factors for radial artery pseudoaneurysm include systemic anticoagulation, inadequate compression after procedure, infection, and multiple arterial punctures.^3^ Distal radial artery access-site pseudoaneurysms are the subject of rare case reports and have been treated with embolization^4^ and manual compression.^5^ In this case, systemic anticoagulation and early resumption of weightlifting likely contributed to development of the pseudoaneurysm despite initially successful hemostasis with a dedicated distal radial access-site compression device. The size and baseline anticoagulation status limited any anticipated success with conservative management in this case. Percutaneous thrombin injection is a reasonable option in select cases with appropriate pseudoaneurysm neck anatomy. In this case, we were concerned that the neck was insufficient for safe thrombin injection. Moreover, his localized pain and sensory changes reflected mass effect from the pulsatile radial pseudoaneurysm and mandated open repair to decompress the area. His pseudoaneurysm was ultimately resected. The distal location of the involved radial artery was amenable to simple resection and distal ligation without interruption of anticoagulation. The dominant ulnar artery provided uninterrupted multiphasic arterial flow to the distal extremity.

## Conclusion

In this article, we present a case of left distal radial artery access-site pseudoaneurysm arising in a patient receiving anticoagulation therapy, which was successfully treated with an open surgical repair.
